# Machine learning models for predicting risk of depression in Korean college students: Identifying family and individual factors

**DOI:** 10.3389/fpubh.2022.1023010

**Published:** 2022-11-17

**Authors:** Minji Gil, Suk-Sun Kim, Eun Jeong Min

**Affiliations:** ^1^College of Nursing, Ewha Womans University, Seoul, South Korea; ^2^Ewha Research Institute of Nursing Science, Ewha Womans University, Seoul, South Korea; ^3^Department of Medical Life Sciences, School of Medicine, The Catholic University of Korea, Seoul, South Korea

**Keywords:** machine learning, depression, college student, family, risk factors

## Abstract

**Background:**

Depression is one of the most prevalent mental illnesses among college students worldwide. Using the family triad dataset, this study investigated machine learning (ML) models to predict the risk of depression in college students and identify important family and individual factors.

**Methods:**

This study predicted college students at risk of depression and identified significant family and individual factors in 171 family data (171 fathers, mothers, and college students). The prediction accuracy of three ML models, sparse logistic regression (SLR), support vector machine (SVM), and random forest (RF), was compared.

**Results:**

The three ML models showed excellent prediction capabilities. The RF model showed the best performance. It revealed five significant factors responsible for depression: self-perceived mental health of college students, neuroticism, fearful-avoidant attachment, family cohesion, and mother's depression. Additionally, the logistic regression model identified five factors responsible for depression: the severity of cancer in the father, the severity of respiratory diseases in the mother, the self-perceived mental health of college students, conscientiousness, and neuroticism.

**Discussion:**

These findings demonstrated the ability of ML models to accurately predict the risk of depression and identify family and individual factors related to depression among Korean college students. With recent developments and ML applications, our study can improve intelligent mental healthcare systems to detect early depressive symptoms and increase access to mental health services.

## Introduction

There is a growing concern about the high prevalence of depression among college students nationwide. According to a systematic review, nearly one-third of college students have experienced depressive symptoms, compared with 9% of the general population (1). In Korea, young adults aged 19–29 had the highest prevalence of depression (25.33%), followed by those aged 30–39 (24.16%), 40–49 (18.67%), 50–59 (18.67%), and 65 and older (13.24%) (2). The first signs of depression in college will have a significant impact on academic success and social relationships. Additionally, it will increase the risk of psychiatric comorbidity and suicide (3, 4), which is the leading cause of death in young adults (5).

Early detection of depression and treatment referral is crucial for alleviating the serious effects of depression (4). However, owing to the social stigma associated with mental disorders, Korean students are reluctant to seek mental health services, making the detection of clinical depression by psychiatrists rather limited (6). Therefore, using a patient-administered screening tool can help increase the screening rates of college students' depression and consequently help identify and diagnose depression before psychiatric appointments (7).

The Center for Epidemiological Studies-Depression Scale (CES-D) (8) is the most used and validated self-report screening tool for the potential existence of depression across a wide age range (9). The CES-D cutoff of 13 was designed specifically to screen for the risk of depression in the Korean population (10). Furthermore, several researchers have used the CES-D to investigate the risk and protective factors of depression.

The well-established risk factors for depression in college students include biological, psychosocial, and environmental factors (11, 12). Additionally, family factors in Korean culture require particular attention because the relationship between parents and children is highly valued owing to the practice of Confucianism (13), which has been influencing hierarchical relationships between parents and children. Korean children are expected to obey their parents, and Korean parents are more involved in the lives of their children than American parents (14, 15).

Numerous studies have shown that family dynamics affect the susceptibility of college students to depression and its persistence (16–19). Kim et al. (17) examined the relationship between the depression of parents and that of their children. Children who perceive overly strict parenting are more likely to experience depression than those who perceive optimal parenting (18). Additionally, a systematic review revealed that parental cancer affects the stress and anxiety levels of children (19). However, most studies used traditional statistical methods, such as logistic regression, which heavily rely on the perspectives of the researchers. Researchers manually choose several variables relevant to a single model and sequentially analyze the relationships between them (20).

With recent advancements in technology and data science, artificial intelligence (AI), including ML techniques, has provided advanced analysis methods for developing prediction systems (20–22). These ML techniques enable more accurate classification and prediction by analyzing complex interacting associations among multiple datasets. However, few studies have applied ML algorithms to predict depression in older adults and depressive relapse in bipolar patients (21, 23). Furthermore, limited studies have explored the family-related risk factors for depression in the Korean family triad of fathers, mothers, and college-aged children. Therefore, this study investigated the performance of different ML algorithms, including random forest (RF), support vector machines (SVM), and sparse logistic regression (SLR), to construct a predictive model in which the algorithm accurately predicts the risk of depression in the family triad dataset. Additionally, the best set of variables associated with the risk of depression among Korean college students was identified using SLR.

## Methods

### Data and sample

This study used family data from a larger study that examined family and individual factors related to depression in the families of Korean college students. An earlier study (17) reported the intergenerational transmission of spirituality and its relationship to depression in the families of Korean college students using only the Spiritual Perspective Scale (SPS) (24), Self-Transcendence Scale (STS) (25), and CES-D (8). This study used all study variables and focused on the analysis results of the ML models to develop the best predictive model by identifying the best set of variables using 171 family data (513 individuals).

The family dataset consisted of families of college students, that is, father, mother, and children triads. The inclusion criteria for families were as follows: (a) must be older than 18, (b) must have a college student, (c) must have signed consent forms to participate from all family members, and (d) must be able to read Korean. Families with members suffering from mental illnesses were excluded.

Participants were recruited from universities and religious institutions (churches and temples) using flyers. They were informed that they would independently, and without interacting with family members, complete the questionnaires. To maintain data independence, they each sealed their completed questionnaire in an envelope. Of 197 families, 26 (13.2%) were excluded because outcome-related variables were missing.

### Outcome variable

The outcome variable was the depression score of college students, which was measured using the CES-D (8). Each item is rated on a 4-point Likert scale (0–3), and the total score ranges from 0 to 60, with a higher score indicating more symptoms of depression. Based on the CES-D cutoff score ≥13 for Koreans (10), we divided the college students into two groups: normal (*n* = 96) and at risk of depression (*n* = 75).

### Predictor variables

The predictor variables consisted of a set of demographic, health, and study variables that were selected based on literature reviews of the risk and mitigating factors for depression among Korean college students (Table 1). Study variables included the Big Five Personality inventory (BFI-10) (26), SPS (24), STS (25), relationship questionnaire (27), Kansas Marital Satisfaction Scale (28), parental bonding instrument (29), and Family Adaptability and Cohesion Evaluation Scale IV (FACES IV) (30).

**Table 1:** Study variables.

**Category**	**Variable**
Descriptive	Age, sex
Education	Background, period, grade
Religion	
Family	Length of marriage (parents), the number of family members, number of offspring or siblings, spending time with family
Income satisfaction	
Social group	Participation in social groups, satisfaction with social group
Severity of disease	Gastrointestinal disease, arthritis, hypertension, diabetes mellitus, kidney disease, cancer, cardiovascular disease, respiratory diseases, ophthalmic diseases, hearing impairment, stroke, psychiatric disorder, other diseases
Sleep quality	
Perceived health status	Mental health, physical health
Physical activity	Aerobic exercise, anaerobic exercise
Alcohol use	
Smoking status	
Big five personality inventory	Extraversion, agreeableness, conscientiousness, neuroticism, openness
Spiritual perspective scale	
Self-transcendence scale	
Relationships questionnaire	Secure attachment, dismissing-avoidant attachment, preoccupied attachment, fearful-avoidant attachment
Kansas marital satisfaction scale (parents)	
Parental bonding instrument (children)	Care, overprotection
Family adaptation and cohesion scales IV	Family cohesion, family flexibility

### Statistical analysis

#### Data description and ML models considered

Table 2 shows the descriptive statistics of the samples by the response variable. According to the variable type, a *t*-test or chi-square test was used, and *p*-values were suggested. To build a prediction model, we considered three ML techniques: SLR, SVM, and RF. Logistic regression is one of the most widely used techniques for predicting binary responses. However, it cannot handle high-dimensional data with a small sample size compared with the number of predictor variables (31). We used the least absolute shrinkage and selection operator (LASSO) penalty to overcome this problem and benefit from variable selection (32). Logistic regression with a LASSO penalty (SLR) considers all variables as inputs and makes some coefficients zero while iterating the optimization procedure. Therefore, this algorithm performs a variable selection procedure because the final model has only a few non-zero coefficients and most variables have zero coefficients. This ensures that the algorithm develops an effective prediction model without experiencing high data dimensionality (31). SVMs are among the most well-known ML techniques for binary classification problems. The SVM searches for a hyperplane in high-dimensional space to effectively segregate data (33). RF is a representative ensemble technique for classification, which collects several fitted results from multiple constructed decision trees and outputs the result voted from most trees (34).

**Table 2:** Demographic characteristics of college students.

		**College students' depression**		***p*** **-Value**
		**Normal**	**Risk**	
		**(*n* = 96, 56.1%)**	**(*n* = 75, 43.9%)**	
**Gender**				
Male	*n* (%)	43 (44.8%)	25 (33.3%)	0.133
Female	*n* (%)	53 (55.2%)	50 (66.7%)	
**Age**				
Fathers	Median [1st, 3rd quartile]	53.00 [50.00, 56.00]	55.00 [52.00, 56.25]	0.053
Mothers	Median [1st, 3rd quartile]	51.00 [48.00, 53.00]	51.00 [49.00, 54.00]	0.615
College students	Median [1st, 3rd quartile]	22.00 [21.00, 24.00]	22.00 [21.00, 23.00]	0.287
**Education**				
Fathers	Median [1st, 3rd quartile]	16.00 [14.00, 18.00]	16.00 [12.00, 18.00]	0.730
Mothers	Median [1st, 3rd quartile]	16.00 [12.00, 16.00]	15.50 [12.00, 16.00]	0.468
**Length of marriage**				
Fathers	Median [1st, 3rd quartile]	24.00 [23.00, 27.00]	24.00 [23.00, 27.00]	0.694
Mothers	Median [1st, 3rd quartile]	25.00 [22.00, 27.00]	24.00 [23.00, 27.00]	0.658
**Spending time with family (hour per week)**				
Fathers	Median [1st, 3rd quartile]	3.00 [1.00, 5.00]	1.75 [1.00, 4.00]	0.045
Mothers	Median [1st, 3rd quartile]	4.00 [2.00, 7.50]	3.25 [1.00, 8.00]	0.668
College students	Median [1st, 3rd quartile]	3.00 [1.00, 8.00]	2.00 [1.00, 5.00]	0.052
**Income satisfaction**				
**Fathers**				
Dissatisfaction	*n* (%)	25 (26.1%)	32 (42.7%)	0.048
Neutral	*n* (%)	51 (53.1%)	27 (36.0%)	
Satisfaction	*n* (%)	20 (20.8%)	16.0 (21.3%)	
**Mothers**				
Dissatisfaction	*n* (%)	30 (31.3%)	32 (42.7%)	0.271
Neutral	*n* (%)	46 (47.9%)	32 (42.7%)	
Satisfaction	*n* (%)	20 (20.8%)	11 (14.6%)	
**College students**				
Dissatisfaction	*n* (%)	17 (17.7%)	30 (40.0%)	0.005
Neutral	*n* (%)	49 (51.1%)	31 (41.3%)	
Satisfaction	*n* (%)	30 (31.2%)	14 (18.7%)	

#### Model tuning

To assess the prediction accuracy of the three models, we divided the dataset into training (70%) and testing (30%) data. For model construction, each of the three considered models had parameters to be tuned to achieve the best performance. We used the “cv.glmnet” function implemented in the “glmnet” R package for tuning the sparsity parameter of logistic regression (35). To reduce bias caused by penalization, we conducted a refitting procedure using ordinary logistic regression. For SVM model tuning, we used the “tune.svm” function of the “e1071” R package (36), which performs a grid search to identify the optimal pair of parameters. Additionally, the RF parameters were tuned using the grid search method. For this purpose, we used the “train” function of the “caret” R package (37).

#### Model comparison

After model fitting, several metrics were computed to compare the prediction accuracies of the three models. We computed the accuracy, positive predictive value (PPV), sensitivity, specificity, F1 score, and AUC. We also reported the estimated coefficients and corresponding *p*-values from an SLR model and variables with a mean decrease Gini greater than 1 to identify the important factors from the RF model for understanding depression in college students.

All hypothesis tests were two-sided, and the statistical significance level was set at *p* < 0.05. All analyzes were conducted using statistical software R (version 4.2.0; R Foundation).

## Results

Descriptive statistics were computed to compare the characteristics between the two groups. The results are shown in Table 2. The *p*-values were computed using the *t*-test or chi-square test depending on the type of each variable. The results demonstrated that the income satisfaction of fathers and college students was significantly different between the normal and depression-risk groups.

Table 3 shows various performance measure values from the three models used for the test dataset. The RF model shows the best overall performance with the exception of AUC. The SVM also demonstrated superior performance compared with SLR, with the exception of AUC. Although other metrics imply that SLR is inferior to the other two techniques, it performs the best in terms of AUC. This finding implied that SLR outperforms other methods in terms of ranking data, even though the thresholding value (0.5) is not optimal for class prediction in this application.

**Table 3:** Performance of machine learning algorithms.

	**Accuracy**	**Precision**	**Sensitivity**	**Specificity**	**F1 score**	**AUC**
Sparse logistic	0.7843	0.7143	0.7500	0.8065	0.7317	0.9032
SVM	0.8039	0.7500	0.7500	0.8387	0.7500	0.7944
Random forest	0.8627	0.8059	0.8500	0.8710	0.8274	0.8605

**Figure 1:**
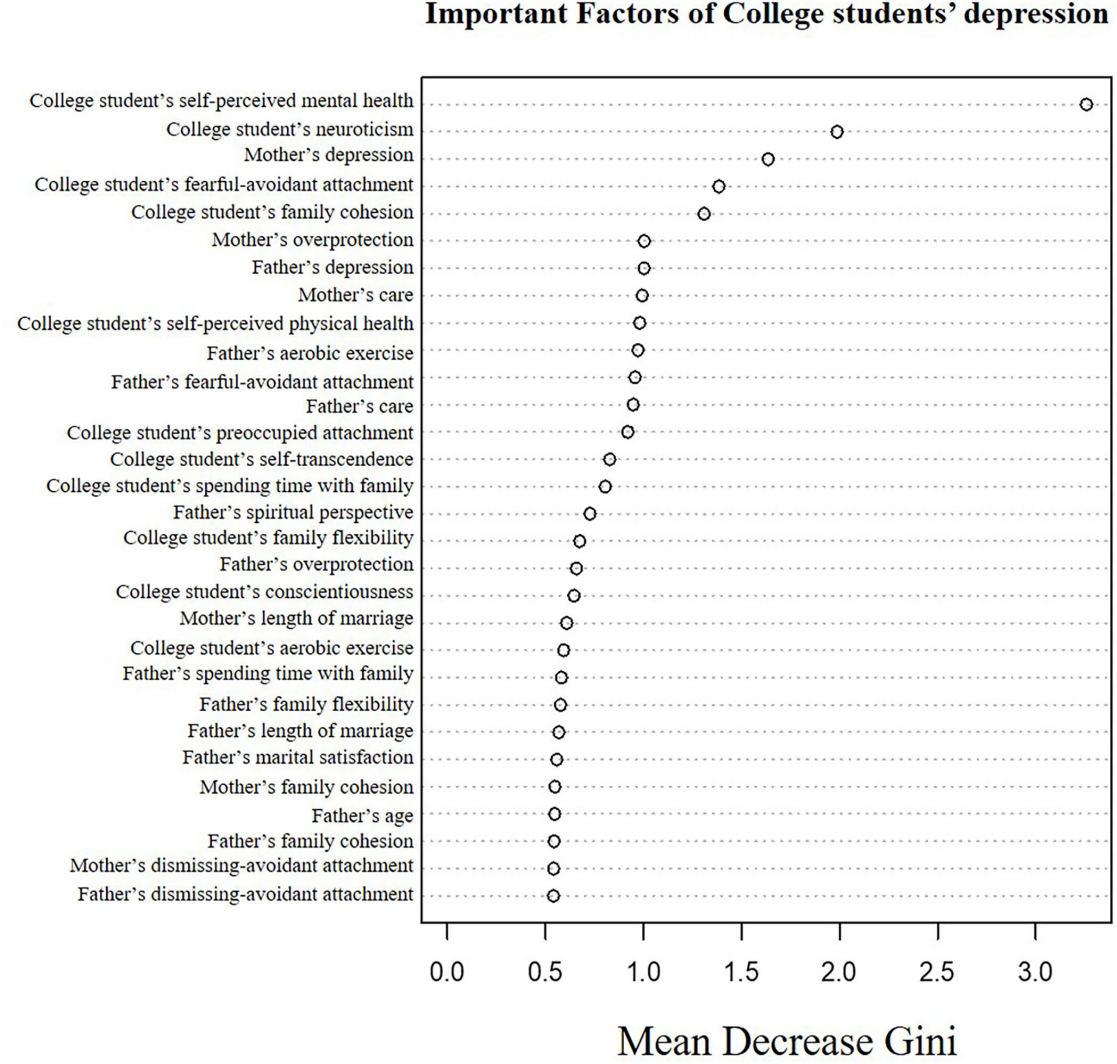
Predictors of college student's depression.

Table 4 shows a final logistic regression fit for selected variables using SLR, and Figure 1 shows significant RF variables. The self-perceived mental health statuses of college students were the most significant variable in the logistic model for understanding and predicting their depression risk (*p* < 0.001). The depression of college students was negatively associated with their self-perceived mental health status [odds ratio (OR) 0.093, 95% CI 0.021–0.272], implying that college students with healthier mental health had a lower risk of depression. According to RF, the self-perceived mental health statuses in college students were the most significant variable for predicting their depression risk. Further analysis using logistic regression showed that fathers with cancer and conscientiousness of college students had a negative relationship with the depression risk in college students, whereas respiratory diseases of mothers and neuroticism of college students had a positive relationship with depression risk. Factors related to college students (conscientiousness and neuroticism of college students) were identified in the final logistic model and were also selected as important features in the RF. Several variables selected by the sparse logistic model are also shown in the list of important variables in the RF model.

**Table 4:** Logistic regression analysis results.

	**Estimate**	**Std error**	**Exp coef**	**Low CL**	**Upper CL**	***p*** **-Value**
Father's severity of cancer	−1.646	0.704	0.193	0.041	0.675	0.019
Father's aerobic exercise	−0.283	0.228	0.753	0.462	1.147	0.213
Mather's severity of cardiovascular disease	0.770	0.591	2.159	0.751	8.154	0.193
Mather's severity of respiratory diseases	1.216	0.520	3.373	1.345	11.313	0.019
College student's self-perceived mental health	−2.377	0.638	0.093	0.021	0.272	0.000
Father's fearful-avoidant attachment	0.496	0.288	1.643	0.958	3.024	0.085
Mather's depression	−0.016	0.037	0.984	0.914	1.060	0.660
College student's conscientiousness	−1.337	0.610	0.263	0.071	0.805	0.028
College student's neuroticism	1.391	0.519	4.021	1.562	12.420	0.007
College student's fearful-avoidant attachment	0.330	0.219	1.391	0.914	2.183	0.131
Mother's care	−0.079	0.072	0.924	0.797	1.060	0.271
College student's income satisfaction-neutral						
College student's income satisfaction-dissatisfaction	1.666	0.859	5.290	1.069	33.254	0.053
College student's income satisfaction-satisfaction	0.831	0.965	2.295	0.351	16.548	0.389

## Discussion

The study provides evidence that the performance of ML algorithms can accurately predict college students who are at risk of depression in the context of parent-child relationships. The ML algorithm can help reduce bias and improve disease prediction accuracy using training datasets to train and test datasets to test (38, 39). The main strength of this study was the use of the family dataset to predict and identify family factors of depression in college students using ML techniques. With recent advancements and applications of ML, our study can help improve intelligent mental healthcare systems to detect early depressive symptoms and increase access to mental health services for college students.

Our data are high-dimensional, where variables are more than samples. This data feature causes two problems. First, the estimates are poorly defined when using linear regressions. Second, despite having analysis results, the interpretation is difficult owing to numerous variables. This dataset feature enabled us to investigate additional analysis techniques, such as SLR, RF, and SVM.

Sparse multivariable logistic regression is a well-known ML technique that enables the application of multivariable logistic regression to high-dimensional data (31). This technique simultaneously conducts estimation and variable selection to obtain interpretable results with estimated effect sizes. Conversely, SVM and RF have been unaffected by the high dimensionality of the data. Recently, these techniques have been widely used to analyze high-dimensional data, and numerous studies showed that these techniques outperform regression models (21, 40). Both techniques use all available variables to develop a prediction model; however, their results are not easily interpretable as those of regression models, which list important variables along with their estimated effect size (41). However, the RF model provides a list of important variables based on how each variable is used by the algorithm.

Our study demonstrated that SVM and RF outperformed sparse multivariable logistic regression in terms of prediction. Most of the metrics from these two methods were superior to those from the sparse multivariate logistic model. Particularly, RF demonstrated the best prediction performance. However, important variables can be identified from the results of sparse multivariable logistic regression, and its prediction performance is comparable with that of SVM and RF. This technique automatically selects significantly effective features using an algorithmic approach and estimates the effect size. Multiple model-selected variables are also included in the list of variables where an RF is suggested as an important feature. This finding suggested that considering the strategic use of multiple ML techniques from both prediction and inference standpoints is important.

In our study, mother's depression, respiratory diseases, and father's cancer, were identified as three family factors significant in predicting depression risk in college students. Logistic regression analysis demonstrated that college students whose mothers had depression and respiratory diseases were more likely to experience depression. However, college students with fathers who have cancer have a lower risk of depression. These findings can be explained by the intergenerational effects of the parent's health status on depression in children. Children may feel stressed, powerless, and depressed in the presence of their mother's depression and fatigue leading to cough and breathlessness (42).

Moreover, several studies have demonstrated that maternal depression increases the risk of depression in early adulthood through poor parenting. Depressed mothers are more likely to express negative emotions toward their children and interact negatively with them, which interferes with the bond between the mothers and their children (43–45). Children with insecure attachments frequently struggle to identify or control their emotions, which may result in depression (46). To successfully reduce the emotional problems of college students and enhance positive mother-child relationships, family-based early interventions must be developed.

However, we found that college students whose fathers had cancer decreased the risk of depression. This is incongruent with a previous study, which found that children whose parents had cancer experience higher levels of depression than those with cancer-free parents (47). These findings contribute new knowledge to our understanding of the relationship between paternal cancer and depression in children. One justification is that fathers who realize more value in spending time with their children than at work after being diagnosed with cancer may increase communication with them, which helps children develop resilience and prevents depression (48, 49). Another justification is that children build resilience and reduce depression when they positively deal with a stressful situation, such as a father being diagnosed with cancer, by interpreting the situation positively and creating meaning (50–52). Future research should focus on understanding the mechanisms of paternal cancer in children with depression. Depression in college students can be measured against different mechanisms of maternal and paternal psychological and mental disorders.

We discovered that five individual factors were important in predicting depression in college students: family cohesion, fearful-avoidant attachment, neuroticism, conscientiousness, and self-perceived mental health. Family cohesion and fearful-avoidant attachment were found to be important factors that contribute to determining depression in college students using the RF approach. According to earlier studies, college students who reported a high level of family cohesion had a lower risk of depression than those with lower levels of cohesion. The former group experienced comfort, support, and togetherness within their families (53–55). However, students with fearful-avoidant attachment, high anxiety about rejection, and avoidance of intimacy experienced more depression than those with secure attachment (56). Because students with fearful-avoidant attachments perceive low family cohesion, repairing parent-child attachments reduces depression by building trust and safety with parents and using them as emotional supporters (55, 57, 58).

Logistic regression results demonstrated that neuroticism increased the risk of depression among college students, whereas conscientiousness decreased this risk. Neuroticism and conscientiousness, which are human personality traits, are associated with depression (59, 60). College students with higher neuroticism who tend to be emotionally unstable might often focus on their negative emotions, whereas those with higher conscientiousness, who tend to be goal-oriented, careful, and efficient, could efficiently direct their attention away from negative emotions (60, 61). Personality traits are difficult to change; however, training college students with neuroticism in adaptive emotional regulation strategies should help them prevent depression.

Our results implied that self-perceived mental health is a significant predictor of depression. The perception of mental health of an individual typically indicates their self-perceived mental health. These results were consistent with a previous study that demonstrated that poor self-perceived health is a risk factor for depression (62). College students who negatively perceive their mental health believe that they need professional treatment, but they refuse it because they believe that seeking help is a sign of weakness (63). However, if college students have good mental health, they do not feel the need to visit the hospital (64). Therefore, it is important to objectively evaluate or detect signs of depression in the mental health of college students.

Our study investigated the performance of the three ML algorithms and identified family and individual factors that could help predict depression in college students. Using AI technologies could reduce depression risk in college students and improve mental health. Our ML algorithms could use family data to screen college students for depression. Healthcare providers can use these ML algorithms to identify college students who may be at risk of depression and help them with early intervention.

This study has some limitations. First, although three factors, mother's depression, college students' cohesion, and college students' fearful-avoidant attachment, were identified as important factors in the RF approach, the direction of the effects of these factors is unknown. Second, it was difficult to interpret the causal relationship between the important factors and depression using the cross-sectional data. Finally, although the severity of chronic disease was included in the variables, it was evaluated from a subjective perspective. This should be considered in future studies.

## Conclusion

This study demonstrated how to use family data to predict depression in college students using the ML models. We analyzed three ML models: sparse multivariable logistic regression, RF, and SVM to predict depression among college students and confirmed the significant contributing factors. The RF model demonstrated the best prediction performance among the three ML models. Additionally, sparse multivariable logistic regression was used to identify the important variables. The RF and sparse multivariable logistic regression results demonstrated that the health status of the parents, family factors of college students, personality traits, and self-perceived mental health were significant factors. Our study can help develop better ML models for mental health that can detect depression in college students.

## Data availability statement

The raw data supporting the conclusions of this article will be made available by the authors, without undue reservation.

## Ethics statement

The studies involving human participants were reviewed and approved by Institutional Review Board of Ewha Womans University. The patients/participants provided their written informed consent to participate in this study.

## Author contributions

MG, S-SK, and EJM: study and manuscript conceptualization, contributed to the discussion, methods, and results. MG and S-SK: contributed to the backgrounds. All authors contributed to the article and approved the submitted version.

## Funding

This study was supported by Basic Science Research Program through the National Research Foundation of Korea (NRF) funded by the Ministry of Science, ICT and Future Planning (Nos. NRF 2022R1A2C2004867 and 2021R1F1A1058613).

## Conflict of interest

The authors declare that the research was conducted in the absence of any commercial or financial relationships that could be construed as a potential conflict of interest.

## Publisher's note

All claims expressed in this article are solely those of the authors and do not necessarily represent those of their affiliated organizations, or those of the publisher, the editors and the reviewers. Any product that may be evaluated in this article, or claim that may be made by its manufacturer, is not guaranteed or endorsed by the publisher.
